# Combined miRNA and SERS urine liquid biopsy for the point-of-care diagnosis and molecular stratification of bladder cancer

**DOI:** 10.1186/s10020-022-00462-z

**Published:** 2022-04-01

**Authors:** Tudor Moisoiu, Mihnea P. Dragomir, Stefania D. Iancu, Simon Schallenberg, Giovanni Birolo, Giulio Ferrero, Dan Burghelea, Andrei Stefancu, Ramona G. Cozan, Emilia Licarete, Alessandra Allione, Giuseppe Matullo, Gheorghita Iacob, Zoltán Bálint, Radu I. Badea, Alessio Naccarati, David Horst, Barbara Pardini, Nicolae Leopold, Florin Elec

**Affiliations:** 1 Clinical Institute of Urology and Renal Transplantation, 400006 Cluj-Napoca, Romania; 2 Iuliu Hatieganu University of Medicine and Pharmacy, 400012 Cluj-Napoca, Romania; 3 Biomed Data Analytics SRL, 400696 Cluj-Napoca, Romania; 4 Institute of Pathology, Charité-Universitätsmedizin Berlin, corporate member of Freie Universität Berlin, Humboldt-Universität Zu Berlin and Berlin Institute of Health, 10117 Berlin, Germany; 5 German Cancer Consortium (DKTK), Partner Site Berlin, and German Cancer Research Center (DKFZ), Heidelberg, Germany; 6 Faculty of Physics, Babeș-Bolyai University, 400084 Cluj-Napoca, Romania; 7 Department of Medical Sciences, University of Turin, 10126 Turin, Italy; 8 Department of Clinical and Biological Sciences, University of Turin, Regione Gonzole, 10, 10043 Orbassano, Italy; 9 Faculty of Biology, Babeș-Bolyai University, 400015 Cluj-Napoca, Romania; 10 Octavian Fodor Regional Institute of Gastroenterology and Hepatology, 400162 Cluj-Napoca, Romania; 11 Candiolo Cancer Institute-FPO IRCCS, 10060 Candiolo, Turin, Italy; 12 Italian Institute for Genomic Medicine (IIGM), IRCCS Candiolo, 10060 Candiolo, Turin, Italy

**Keywords:** Bladder cancer, microRNA, SERS, Liquid biopsy, Molecular subtypes, Biomarkers

## Abstract

**Background:**

Bladder cancer (BC) has the highest per-patient cost of all cancer types. Hence, we aim to develop a non-invasive, point-of-care tool for the diagnostic and molecular stratification of patients with BC based on combined microRNAs (miRNAs) and surface-enhanced Raman spectroscopy (SERS) profiling of urine.

**Methods:**

Next-generation sequencing of the whole miRNome and SERS profiling were performed on urine samples collected from 15 patients with BC and 16 control subjects (CTRLs). A retrospective cohort (BC = 66 and CTRL = 50) and RT-qPCR were used to confirm the selected differently expressed miRNAs. Diagnostic accuracy was assessed using machine learning algorithms (logistic regression, naïve Bayes, and random forest), which were trained to discriminate between BC and CTRL, using as input either miRNAs, SERS, or both. The molecular stratification of BC based on miRNA and SERS profiling was performed to discriminate between high-grade and low-grade tumors and between luminal and basal types.

**Results:**

Combining SERS data with three differentially expressed miRNAs (miR-34a-5p, miR-205-3p, miR-210-3p) yielded an Area Under the Curve (AUC) of 0.92 ± 0.06 in discriminating between BC and CTRL, an accuracy which was superior either to miRNAs (AUC = 0.84 ± 0.03) or SERS data (AUC = 0.84 ± 0.05) individually. When evaluating the classification accuracy for luminal and basal BC, the combination of miRNAs and SERS profiling averaged an AUC of 0.95 ± 0.03 across the three machine learning algorithms, again better than miRNA (AUC = 0.89 ± 0.04) or SERS (AUC = 0.92 ± 0.05) individually, although SERS alone performed better in terms of classification accuracy.

**Conclusion:**

miRNA profiling synergizes with SERS profiling for point-of-care diagnostic and molecular stratification of BC. By combining the two liquid biopsy methods, a clinically relevant tool that can aid BC patients is envisaged.

**Supplementary Information:**

The online version contains supplementary material available at 10.1186/s10020-022-00462-z.

## Introduction

Bladder cancer (BC) is one of the most common malignant tumors, with approximately 549.000 new cases and 200.000 deaths annually, 70% of them being non-muscle invasive (NMIBC) at the initial diagnosis (Saginala et al. 2020). BC has the highest per-patient cost (Mossanen and Gore 2014), mainly because BC diagnosis, follow-up and risk stratification rely on pathology reports and multiple cystoscopies, an invasive and resource-consuming strategy.

BC patients usually present with lower urinary tract symptoms or hematuria, prompting immediate cystoscopy for confirming the malignant cause. The bladder tumor is then resected using transurethral resection and sent for pathology analysis, which allows the staging in NMIBC or muscle-invasive BC (MIBC). Unfortunately, the predictive power of the current histological classification of BC is suboptimal, failing to account for the molecular underpinnings of BC (Kardoust Parizi et al. 2021). Molecular classification of BC in two major subtypes with different prognostic significance has recently been proposed: (i) a basal subtype with a squamous differentiation pattern and a more aggressive disease, and (ii) a luminal subtype with a milder prognosis (Choi et al. 2014; Sjodahl et al. 2013; Cancer Genome Atlas Research N 2014; Rodriguez Pena et al. 2019). However, similarly to the histological classification, the stratification of BC into basal and luminal subtypes is possible only using RNA sequencing or immunohistochemistry analysis of tissue samples obtained during endoscopic procedures (Kouba et al. 2020). Depending on the results of the pathology analysis, the recommended treatment can include either cystectomy, usually for MIBC, or transurethral resection of bladder tumor followed by lifelong follow-up by cystoscopy, usually for NMIBC (Babjuk et al. 2019).

There has been a continuous quest to develop liquid biopsy tools to overcome the need for invasive and costly endoscopic procedures in BC diagnosis, follow-up, and molecular stratification. For instance, microRNAs (miRNAs), short transcripts that fine-tune gene expression at the post-transcriptional level (Dragomir et al. 2018, 2021; Gebert and MacRae 2019), have been extensively explored as means to perform liquid biopsy (Matullo et al. 2016). We have previously demonstrated that using miR-30a-5p, let-7c-5p, miR-486-5p, and smoking status, it is possible to diagnose BC with an area under the curve (AUC) of 0.70 (Pardini et al. 2018). However, after almost two decades of miRNA research in oncology, no miRNA-based diagnostic tool has been approved. We believe that ingenious strategies need to be developed to integrate miRNAs into clinical practice.

Surface-enhanced Raman spectroscopy (SERS) has recently tackled the complex task of performing liquid biopsy in cancer (Guerrini and Alvarez-Puebla 2019). SERS refers to the use of plasmonic substrates, most commonly silver and gold colloids, to amplify the Raman signal of molecules adsorbed onto the metal surface (Bonifacio et al. 2015; Moisoiu et al. 2021). We have previously demonstrated the possibility to use SERS profiling of serum or urine for the diagnosis of both genitourinary cancer (prostate cancer) (Stefancu et al. 2018) and non-genitourinary cancer (breast cancer) (Moisoiu et al. 2019), yielding a diagnostic accuracy in the order of 90%. The most important advantage of SERS is that it provides information concerning the molecular structure of the sample within seconds (purine metabolites, carotenoids etc.) through a simple laser scan, making it an ideal point-of-care diagnostic tool.

Building on previous studies suggesting that both miRNA or SERS profiling represent independent promising strategies for application in liquid biopsy, in this study, we assessed for the first time the synergism between miRNA and SERS profiling of urine for the point-of-care diagnosis and molecular stratification of BC.

## Material and methods

### Patients

We prospectively enrolled 15 patients with BC visiting the Clinical Institute of Urology and Renal Transplantation, Cluj-Napoca, Romania. In parallel, we enrolled 16 controls (CTRL) visiting the same clinic: patients with lower urinary tract symptomatology similar to BC, but with no malignant disease after cystoscopy plus pathology analysis (Additional file 1: Table S1). Additionally, any subject with other malignant diseases was excluded from the control patient cohort. The study was approved by the Ethics Committee of the Clinical Institute of Urology and Renal Transplant—No. 1/2018. For all these patients, 40 ml of fresh urine samples were collected in standard tubes and centrifuged at 3600×*g* for 10 min. The supernatant was collected and aliquoted for SERS and miRNA profiling. Additionally, if available, we also collected for research purposes a formalin-fixed paraffin-embedded tissue block for each BC patient.

In order to further validate the miRNAs of interest, we retrospectively analyzed a second cohort (Italian cohort) for which next-generation sequencing (NGS) data of the whole miRNome were already available. The study population consisted of 116 urine samples from men enrolled in the Turin Bladder Cancer Study (TBCS) and fully described in (Pardini et al. 2018; Sabo et al. 2020). In the study were included 66 BC cases and 50 CTRLs (Additional file 1: Table S2). BC patients were all newly diagnosed, histologically confirmed cases of BC registered at two Urology Departments of A.O.U. Città della Salute e della Scienza, in Turin (Italy). CTRLs were males recruited randomly from patients treated at the same urology departments for non-neoplastic disease (prostatic hyperplasia, cystitis, and other) or from patients treated at the medical and surgical departments for hernias, vasculopathy, diabetes, heart failure, asthma, or other benign diseases. Subjects with cancer, liver, or renal diseases, and smoking-related conditions were excluded. Urine was collected from all study participants who signed a written consent to participate in the study according to the Helsinki Declaration. The study was approved by the Interhospital Ethical Board of San Giovanni Battista/C.T.O./C.R.F./Maria Adelaide hospitals (Turin, Italy) and the Institutional Review Boards of the Italian Institute for Genomic Medicine (IIGM). miRNA expression levels measured in urine of the Italian cohort were previously reported in Pardini et al. (2018).

### Immunohistochemistry staining and image acquisition

Formalin-fixed and paraffin-embedded tissue samples of BC were cut into 4 μm sections. For the subsequent immunohistochemical staining, a BenchMark XT immunostainer (Ventana Medical Systems, Tucson, AZ) was used. For antigen retrieval, sections were incubated in CC1 mild buffer (Ventana Medical Systems, Tucson, AZ) for 30 min at 100 °C or in protease 1 for 8 min. The sections were stained with anti-p53 antibody (DO-7, Dako, 1:50), anti-GATA 3 antibody (HG3-31, SantaCruz, 1:50), anti-ER (SP1, Ventana, ready to use), anti-Her2neu (4B5, Ventana, ready to use), anti-CK5/6 (EP24,EP67, abcam, 1:100), anti-CD44 (DF1485, Dako, 1:50), anti-CK20 (KS20.8, Dako, 1:100) and anti-Uroplakin III (AU1, Progen, ready to use) for 60 min at room temperature, and visualized using the avidin–biotin complex method and DAB. A detailed description of the antibodies used for the study can be found in Additional file 1: Table S3. We stained the cell nuclei by additionally incubating for 12 min with hematoxylin and bluing reagent (Ventana Medical Systems, Tucson, AZ).

The stains were evaluated using an Olympus BX50 and Olympus BX46 microscopes (Olympus Europe). Histological images were acquired with the digital slide scanner PANNORAMIC 1000 (3DHISTECH).

### Immunohistochemistry scoring

Two pathologists (MPD and SS) performed all histological analyses, and all scores represent the average of their independent scoring.

For cellular tumor antigen suppressor p53 (TP53) scoring, we used the Allred et al. score (Allred et al. 1998), which was proposed by Stec et al. as a prognostic marker for NMIBC (Stec et al. 2020). Briefly, we analyzed the BC tumor area and scored between 0 and 5 the percentage of stained cell nuclei (proportion score—PS) and between 0 and 3 the intensity of nuclear staining (intensity score—IS). The final score represented the sum of PS and IS. A detailed presentation of the Allred score can be found in Additional file 1: Table S4. We considered a score > 7 (intense nuclear accumulation of TP53 in most tumor cells) or = 0 (complete loss of TP53 in most tumor cells) as TP53 mutated.

Trans-acting T-cell-specific transcription factor GATA-3 (GATA 3); Estrogen Receptor alpha (ER alpha); Keratin, type II cytoskeletal 5 and 6 (CK5/6); Keratin, type I cytoskeletal 20 (CK20); CD44 antigen (CD44); and Uroplakin III staining intensity were evaluated for percentage of extent (0–100%) in the entire tumor area of a slide of interest. Receptor tyrosine-protein kinase erbB-2 (Her2neu) was analyzed using scores established in gastric cancer and BC. Briefly, Her2neu was given a score of 0 if it stained < 10% of tumor cells; 1 + if it stained weak/only one part of the membrane of ≥ 10% of tumor cells; 2 + if it stained moderate/weak the complete/basolateral membrane of ≥ 10% of tumor cells; and 3 + if it stained strong complete/basolateral membrane ≥ 10% of tumor cells (Abrahao-Machado and Scapulatempo-Neto 2016).

We slightly modified the protocol of Rodriguez Pena et al. to classify luminal and basal BC (Rodriguez Pena et al. 2019) by replacing Uroplakin II with Uroplakin III, which is the established uroplakin antigen at our Institute (Kaufmann et al. 2000). Briefly, we divided the tumors into luminal and basal by using CK20 and Uroplakin III scores as surrogates for the luminal subtype and CD44 as well as CK5/6 scores as surrogates for the basal subtype. Each BC was assigned to luminal or basal based on the highest of any of the four surrogate markers (i.e., a BC sample with a CK5/6 score higher than the other three markers was classified as a basal subtype).

### miRNA profiling by next-generation sequencing

The protocol for urine collection, storage, and processing together with library preparation has been previously described elsewhere (Pardini et al. 2018; Ferrero et al. 2018). For both cohorts, the same methodology was used. Briefly, total RNA was extracted from urine supernatant samples (Ferrero et al. 2018) using the Urine microRNA Purification kit (Norgen Biotek, Canada), according to the manufacturer’s standard protocol. RNA quality and quantity were verified according to MIQE guidelines (http://miqe.gene-quantification.info/). For all samples, RNA concentration was quantified by Invitrogen Qubit® 4 Fluorometer with Qubit® microRNA Assay Kit (Invitrogen, Milan, Italy).

Small RNA transcripts were converted into barcoded cDNA libraries with the NEBNext Multiplex Small RNA Library Prep Set for Illumina (New England BioLabs, USA) and run on Illumina NextSeq 500 platform (Illumina, USA).

Raw reads adapter clipping was performed with the Cutadapt software (version 1.18) (Martin 2011). Reads longer than 14 nucleotides were mapped to a small non-coding RNA (sncRNA) reference with the bwa alignment software (version 0.7.17-r1188) (Li 2012), using the mem algorithm and a seed length of 10. Only alignments without mismatches or indels were considered, and those with the highest quality were used to assign each read to a unique sncRNA. Thus, sncRNAs were quantified for each sample and then merged into a single count matrix, setting missing sncRNAs to zero.

Differential expression analysis was performed with the DESeq2 Bioconductor’s package (version 1.22.2) (Love et al. 2014). For each model, samples with missing covariates were dropped, and only sncRNAs where at least 70% of the remaining samples had counts greater than 5 were tested. sncRNAs were considered significantly associated with a condition or a trend if their p-value, after adjustment for multiple testing by false discovery rate (FDR), was below the 0.05 threshold.

### miRNA RT-qPCR

Candidate miRNA biomarkers were replicated in urine samples using the miRCURY LNA miRNA PCR Assays (Qiagen, Milan, Italy). Reverse transcription (RT) was performed using the miRCURY LNA™ RT kit (Qiagen, Milan, Italy) according to the manufacturer’s instructions. For RT-qPCR, complement DNA (cDNA) was diluted 1:60.

3 μL of 1:60 water-diluted cDNA products were mixed at 5 μL of 2 × miRCURY SYBR Green Master mix with 0.5 μL of ROX Reference dye, and 1 μL of specific miRNA probe (Qiagen). All cDNA products were prepared in triplicate PCR reactions following the manufacturer’s instructions. For quality control purposes, one RNA sample was measured twice, and a sample containing nuclease-free water and carrier RNA was profiled as a negative control. All the reactions were run on an ABI Prism 7900 Sequence Detection System (Applied Biosystems, Foster City, CA, USA), according to the manufacturer`s instructions. A melt curve analysis was performed for amplification specificity of each individual target per sample. The following specific primers from Qiagen miRCURY LNA system were used: hsa-miR-185-5p (#YP00206037), hsa-miR-205-3p (#YP00205602), hsa-miR-210-3p (#YP00204333), hsa-miR-204-5p (#YP00206072), hsa-miR-1246 (#YP00205630, hsa-miR-615-3p (#YP00204453), and hsa-miR-34a-5p (#YP00204486).

The analyses were performed calculating delta Ct (ΔCt) values by global mean normalization (ΔCt = Ct gene—Ct mean of 7 analyzed miRNAs of interest). MiRNAs with a Ct value > 38 were deemed to be not detected. To avoid biased inference due to RT-qPCR non-detects (Ct value = 40), a left-censoring approach was employed. Ct values of 40 were in fact substituted with the highest observed Ct value for a given miRNA (McCall et al. 2014). Finally, the relative expression of each miRNA was calculated using the Equation 2^−ΔCT^.

For determining differently expressed miRNAs, we first assessed whether the data followed a normal distribution using the Shapiro‐Wilk normality test. Then, we identified and excluded outliers using the ROUT method with a Q = 1% (Motulsky and Brown 2006). Finally, for the comparison between groups, p‐values were determined with an unpaired t-test if the data were normally distributed, while the non‐parametric Mann‐Whitney‐Wilcoxon test was applied on tied values with a non‐normal distribution, whereas Kolmogorov–Smirnov test was applied on untied values with a non‐normal distribution (Dragomir et al. 2019).

### SERS profiling

For the SERS analysis, 50 µL of urine was mixed with 450 µL of methanol and centrifuged for 10 min at 5800×*g*. The supernatant was carefully collected in order not to disturb the pellet. The SERS analysis was performed using silver nanoparticles synthesized by reduction with hydroxylamine hydrochloride (hya-AgNPs) (Leopold and Lendl 2003). Nine µL of hya-AgNPs were mixed with 1 µL of centrifuged urine. Then, the hya-AgNPs were activated by adding 1 µL of Ca(NO_3_)_2_ 10^–2^ M (final concentration of Ca^2+^ 10^–3^ M). A drop of 5 µL from this mixture was deposited on a microscope slide covered with aluminum foil, and the SERS spectra were immediately acquired. The experimental setup consisted of a portable Raman spectroscope (iRaman, BW-Tek) equipped with a laser emitting at 532 nm and a 20X (NA 0.4) objective. The background was acquired as a separate spectrum and then subtracted from the SERS spectrum of the samples. Each measurement consisted of an average of 2 acquisitions, 10s each, and was performed with the laser power set to 30% (18 mW).

The SERS spectra were pre-processed using Quasar-Orange software, Orange-Spectroscopy library (Bioinformatics Laboratory of the University of Ljubljana) (Toplak et al. 2021). The pre-processors applied to the SERS spectra presented in this study are similar to the ones employed in other studies and the final aim was to bring SERS liquid biopsy closer to a standardized method. The spectral region between 400 and 1800 cm^−1^ was used for further analysis. Pre-processing consisted of vector normalization, Rubber-band baseline subtraction, and smoothing (Savitzky-Golay, with the window set to 5 and the polynomial order set to 2). To explore the data and to reduce the dimensionality, principal component analysis (PCA) was performed. Then, the first 11 principal components (PCs) (which explain 98% of the initial variance of the data) were ranked based on the difference in score values between the BC and CTRL groups using the p-value yielded by Student’s t-test. A probability p-value of less than 0.05 was considered significant. The score values of statistically significant PCs were then used as input for supervised classification algorithms (naïve Bayes, logistic regression, and random forest).

The SERS spectra of the CTRL group showed bands in the 500–550 cm^−1^ region and an increased background in the 1730–1800 cm^−1^ range; however, no assignment was found for these spectral features. Thus, for basal and luminal BC classification we kept only the 550–1730 cm^−1^ spectral range. We tested the statistical difference of the first 11 PCs (which explain 99% of the initial variance) between basal and luminal groups and PC8 was the only PC with a p-value < 0.05 (Student’s t-test). Hence, the classifiers (naïve Bayes, logistic regression, and random forest) were implemented on PC8 scores.

When comparing high-grade with low-grade BC, none of the PCs reached statistical significance. Thus, all the first 11 PCs (explaining 99% of the initial variance) were used for high-grade and low-grade classification.

### miRNA target enrichment analysis

Functional enrichment analysis of miRNA target genes was performed using RBiomirGS v0.2.12 (Zhang and Storey 2018) considering the set of validated miRNA-target interactions retrieved from miRTarBase v8.0 (Huang et al. 2020) and miRecords (Xiao et al. 2009). The analysis was performed on gene sets from the Molecular Signatures Database v7.4 (Liberzon et al. 2015) for the Gene Ontology Biological Processes (*c5.go.bp.v7.4*), Reactome (*c2.cp.reactome.v7.4*), and KEG (*c2.cp.kegg.v7.4*) gene set libraries. The log2FC and adjusted p-value computed in the differential expression analysis were used as input for the analysis. Gene sets characterized by an FDR-adjusted p-value lower than 0.05 and involving at least two target genes were considered as significantly enriched.

### Statistical analysis

All statistical analyses were performed using GraphPad Prism 8 software and Quasar-Orange software (Bioinformatics Laboratory of the University of Ljubljana) (Toplak et al. 2021).

For logistic regression, the regularization type was set to Lasso and C parameter 80. For random forest, 5 trees were implemented. All the models were cross-validated by leave-one-out (LOO) method. A receiver operating characteristic (ROC) curve of the probabilities predicted by all the classifiers was built. AUC of ROC curve, classification accuracy, F1 score, precision and recall were used to interpret the classifiers capacity to discriminate the groups. Precision (positive predicted values) is the number of true positive results divided by the sum of true and false positive results. Recall (sensitivity) represents the ratio between true positive results and the sum of true positive and false-negative results. F1-score is used in statistical analysis for binary classification and represents the harmonic mean of precision and recall.

$$F1=2\frac{Precision*Recall}{Precision+Recall}$$

Classification accuracy is the mean of sensitivity (true positive rate) and specificity (true negative rate). The quality performance metrics are represented as average of the values from each repetition of the cross validation. For the classification based on SERS and miRNAs, data were normalized to unity prior to the building of the model.

## Results

### Combined urine miRNA and SERS profiling can accurately distinguish patients with bladder cancer from controls

NGS-based miRNome profiling of urine of the prospective cohort yielded expression levels for 200 testable miRNAs between BC and CTRL. Thirty-three differentially expressed miRNAs achieved statistical significance, of which 20 were upregulated, and 13 were downregulated (Additional file 1: Table S5 and Additional file 1: Fig. S1A).

Target gene enrichment analysis yielded a total of 737 significantly enriched (adj. p < 0.05) functional terms (683, 29, and 25 from GO Biological Processes, Reactome, and KEGG, respectively) for the validated targets of differentially expressed miRNAs (Additional file 1: Fig. S1B and Additional file 2: Table S6). Targets of upregulated miRNAs were enriched for processes related to cell death and adipocyte proliferation (Additional file 1: Fig. S1B). Conversely, targets of downregulated miRNAs were enriched in processes related to nucleotide metabolism and development (Additional file 1: Fig. S1B).

**Fig. 1:**
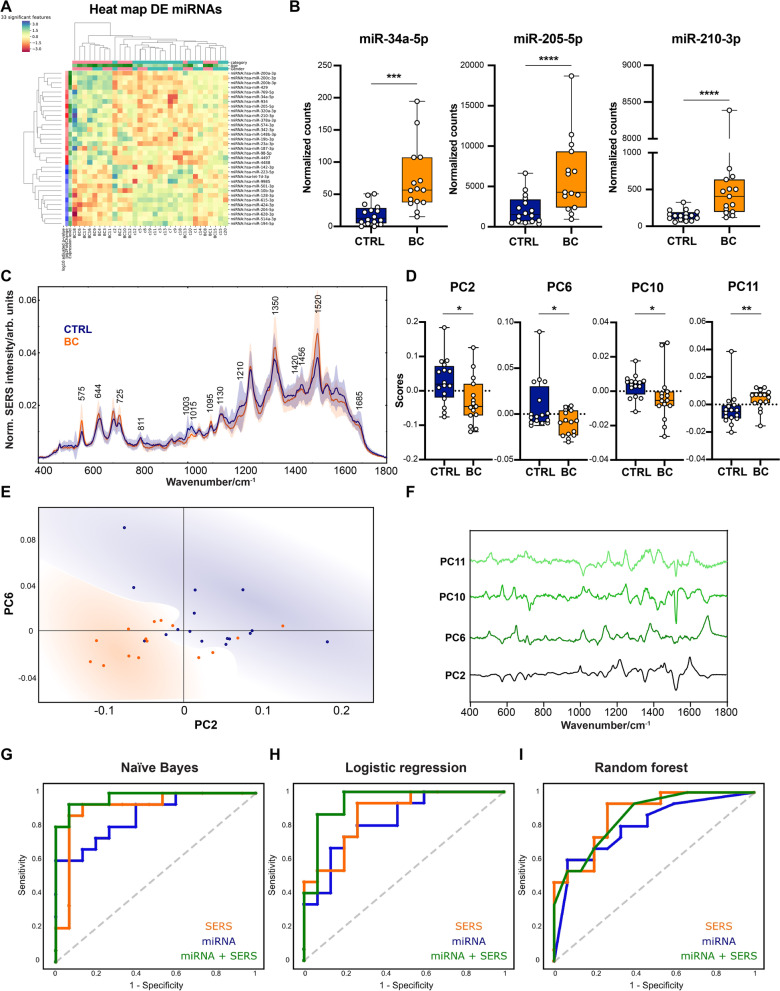
Combined urine miRNA and SERS profiling can accurately distinguish bladder cancer (BC) patients from controls. **A** Heat map of differentially expressed miRNAs by NGS analysis in urine between BC patients and controls (CTRL). The color scale shows the log_10_ of the normalized counts. **B** The top three differentially expressed miRNAs by NGS analysis between BC and CTRL. **C** The average SERS spectra of urine for the BC and CTRL groups (line) and standard deviation (shade). **D** The distribution of score values for principal component (PC) 2, 6, 10, 11 for BC and CTRL. **E** Score plot of PC6 and PC2 for BC and CTRL patients. **F** SERS peaks of PC2, PC6, PC10, and PC11. **G**–**I** Head-to-head comparison of the receiver operating characteristic (ROC) curves for the classification accuracy yielded by miRNA alone, SERS alone, or the combination of the two using three supervised classification algorithms (naïve Bayes (G), logistic regression (H), and random forest (I)). Mean ± SD. *p < 0.05; **p < 0.01; ***p < 0.001; ****p < 0.0001. Abbreviation: DE- differentially expressed

Heat map and unsupervised clustering for the 33 differentially expressed miRNAs are shown in Fig. 1A. The results of the unsupervised clustering showed that there is a tendency for samples to cluster in the BC and CTRL groups. Since we were interested in developing a classifier that can be easily translated into clinical practice, we selected a short set of miRNAs consisting of the top three differentially expressed miRNAs (the most significant adj. p-values and highest fold change) between BC and CTRL: miR-34a-5p, miR-205-5p, and miR-210-3p (Fig. 1B). We confirmed these three miRNAs by RT-qPCR to be overexpressed in BC vs CTRL (Additional file 1: Fig. S1C) and further validated their upregulation by NGS in a large retrospective cohort of BC (n = 66) vs. CTRL (n = 50) (Additional file 1: Fig. S2).

Next, three machine learning algorithms (naïve Bayes, logistic regression, and random forest) were run using the NGS expression data from the prospective cohort on the panel consisting of miR-34a-5p, miR-205-5p, and miR-210-3p, yielding an AUC of 0.87 for naïve Bayes, 0.84 for logistic regression, and 0.81 for random forest (average AUC 0.84 ± 0.03). A detailed report concerning the classification accuracy yielded by the three miRNAs is shown in Additional file 1: Table S7.

In parallel, we also performed SERS profiling of the same urine samples. The average SERS spectra of urine for the BC and CTRL groups are shown in Fig. 1C. The most prominent spectral differences between the two groups regarded SERS bands attributed to purine metabolites (mainly uric acid and hypoxanthine) and creatinine (Additional file 1: Table S8). Higher intensities of the SERS bands at 644, 725, 1350 cm^−1^ (assigned to uric acid and hypoxanthine) in urine samples from cancer patients, compared to controls, were reported earlier (Phyo et al. 2021; Mistro et al. 2015; Iancu et al. 2022). Indeed, higher levels of uric acid and hypoxanthine in samples from cancer patients, compared to controls were expected since cancer is associated with a rise in cellular turnover rate (Ridi and Tallima 2017) and xanthine oxidoreductase, which converts hypoxanthine to xanthine, is downregulated in cancer (Linder et al. 2005). Next, PCA was performed to reduce the data dimensionality. The first 11 PCs, that explained 98.3% of the initial variance, were kept for further analysis (Additional file 1: Fig. S3A, B). Out of the 11 PCs, 4 of them (PC2, PC6, PC10, and PC11) exhibited a statistically significant difference between BC and CTRL groups (Fig. 1D), as revealed by t-test feature selection. Unfortunately, no urine was available for the retrospective cohort to validate the differentially expressed PCs. The score values of PC2 and PC6 indicated a clear tendency for the unsupervised clustering of the two groups (Fig. 1E). Next, the 4 previously selected PCs containing different SERS peaks (Fig. 1F), were employed as input for supervised classification algorithms, yielding an AUC of 0.86 for naïve Bayes, 0.87 for logistic regression, and 0.78 for random forest (average AUC 0.84 ± 0.05) (Additional file 1: Table S7).

Additionally, we sought to explore the synergism between the two orthogonal liquid biopsy strategies. For this, the NGS expression of the three selected miRNAs (miR-34a-5p, miR-205-5p, and miR-210-3p) was combined with the previous four selected PCs, and the supervised machine learning algorithms ran on the set of combined data. Head-to-head comparisons of the classification accuracy yielded by miRNA alone, SERS alone, or the combination of the two showed that the latter achieved the best results across all 3 classification algorithms yielding an average AUC of 0.92 ± 0.06 (AUC of 0.97, 0.94, and 0.86 for naïve Bayes, logistic regression, and random forest, respectively), (Fig. 1G–I and Additional file 1: Table S7).

### Combined urine miRNA and SERS profiling does not predict BC grade but correlates with the molecular classification of BC

Among the 15 patients with BC from the prospective cohort, 8 exhibited high-grade tumors and 7 low-grade tumors (Additional file 1: Fig. S4A). The only differentially expressed miRNA between high-grade and low-grade groups was miR-1246 (Additional file 1: Fig. S4B–D, Additional file 1: Table S9). We further checked the expression of miR-1246 using RT-qPCR and observed its upregulation in high-grade tumors although not reaching statistical significance (Additional file 1: Fig. S4E). In our retrospective cohort consisting of 26 low-grade and 40 high-grade BCs, we detected no difference between the two subgroups (Additional file 1: Fig. S4F). Despite this, miR-1246 being our only candidate, we decided to test its capacity to stratify patients with BC in high- and low-grade.

The average SERS spectra of urine for high- and low-grade BC are shown in Additional file 1: Fig. S4G. The results of the PCA analysis showed that none of the PC achieved statistical significance between the high- and low-grade groups and that there was a weak tendency for the unsupervised clustering of the two groups (Additional file 1: Fig. S4H). As expected, neither miR-1246 NGS expression levels (average AUC of 0.47) nor SERS profiling (average AUC of 0.46) could efficiently predict tumor grade, the combined miRNA and SERS dataset yielding an AUC of 0.39 for logistic regression, 0.64 for naïve Bayes, and 0.62 for random forest (Additional file 1: Fig. S4I–K, and Additional file 1: Tables S10). These data point out how little the tumor grade reflects the phenotype of a tumor.

**Fig. 2:**
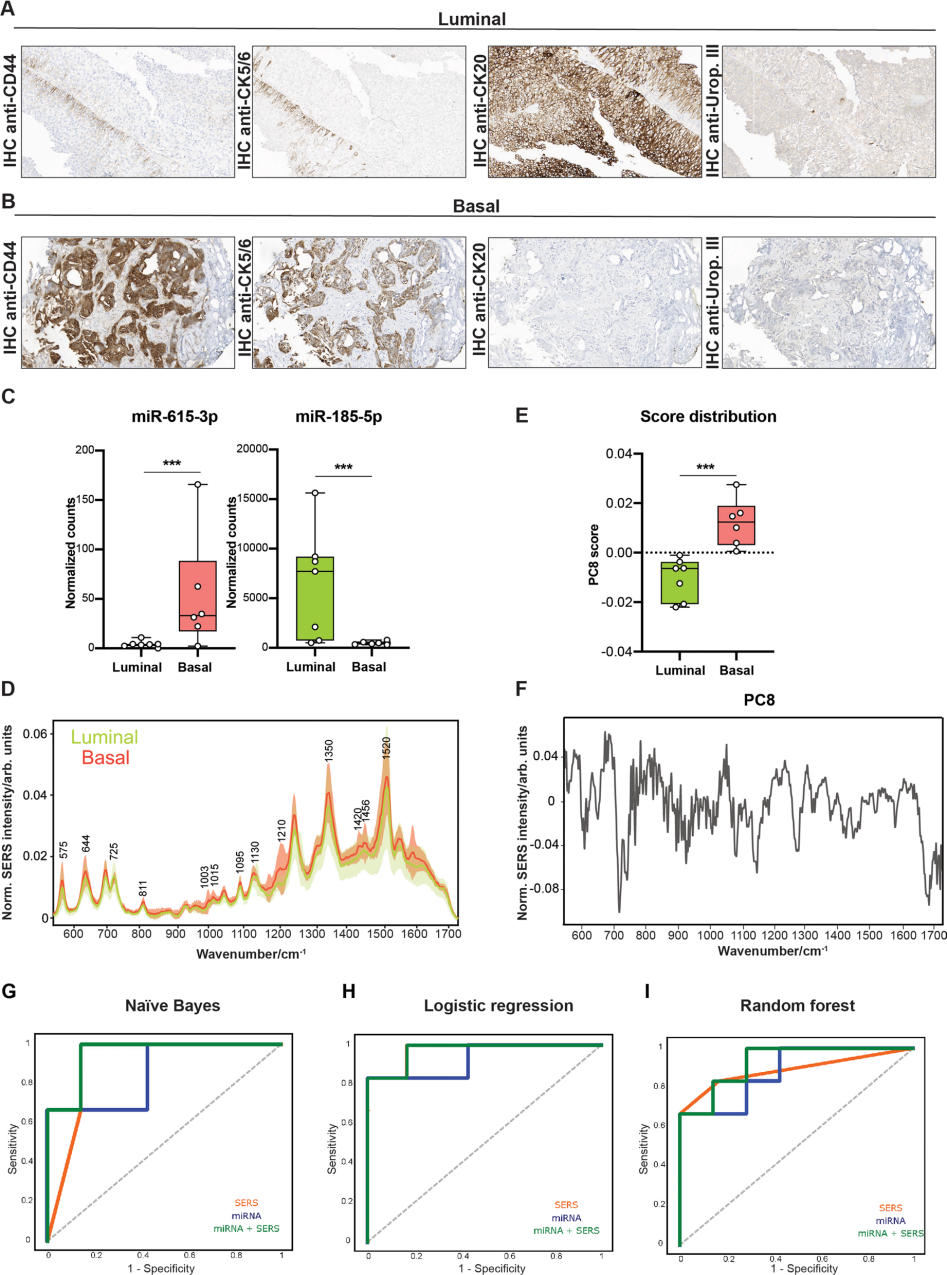
Combined urine miRNA and SERS profiling correlates with the molecular classification of bladder cancer (BC). **A** Immunohistochemical staining aspect of luminal type BC. **B** Immunohistochemical staining aspect of basal type BC. **C** The two differentially expressed miRNAs by NGS analysis between luminal and basal BC. **D** The average SERS spectra of urine for the luminal and basal BC. **E** The score values of principal component (PC) 8 in luminal versus basal BC. **F** Loading plot of PC8. **G**–**I** Head-to-head comparison of the receiver operating characteristic (ROC) curves for the classification accuracy yielded by miRNA alone, SERS alone or the combination of the two using three supervised classification algorithms (naïve Bayes (G), logistic regression (H) and random forest (I)) for luminal and basal BC. Mean ± SD. ***p < 0.001

Thus, we assessed the accuracy of miRNA and SERS profiling of urine in achieving a molecular stratification of the patients. To this aim, we performed immunohistochemistry (IHC) analysis on the available prospective BC samples (n = 13). In regard to TP53 mutation status, only three samples exhibited a TP53 mutated expression pattern (Additional file 1: Fig.S5A). Not surprisingly, two of them had developed MIBC and underwent radical cystectomy. Most of the analyzed tumors showed high GATA3 expression (average GATA3 expression across all samples 93.15 ± 11.32); on the contrary, most tumors lacked ER expression (2.88 ± 5.21). Finally, BCs showed widely distributed Her2neu scores (1.58 ± 1.05, Additional file 1: Fig. S5A and Additional file 1: Table S11). Because of the heterogeneous expression of these markers, none of them could be used to classify BCs. Hence, we further classified BCs into luminal and basal subtypes using an IHC surrogate classification (Rodriguez Pena et al. 2019). Of the 13 BCs, 7 showed a luminal phenotype, with high expression of CK20 and/or Uroplakine III versus CD44 and CK5/6 (Fig. 2A and Additional file 1: Table S11). The other 6 tumors were of basal subtype, showing higher expression for CD44 and CK5/6 (Fig. 2B and Additional file 1: Table S11).

Out of the 202 urinary miRNAs tested by NGS, 25 were differentially expressed between luminal and basal types of BC (Additional file 1: Table S12). The volcano plot of the tested miRNAs and the heat map of the 25 differentially expressed miRNAs are reported in Additional file 1: Fig. S5B, C. The top three differentially expressed miRNAs between luminal and basal groups were miR-615-3p, miR-185-5p (Fig. 2C, Additional file 1: Table S12), and miR-204-5p (Additional file 1: Fig. S5D and Table S12). Next, we checked the expression of these three miRNAs by RT-qPCR and confirmed the upregulation of miR-615-3p and downregulation of miR-185-5p in basal vs. luminal type BC and observed no difference between the two groups for miR-204-5p (Additional file 1: Fig. S5E). Therefore, for the subsequent analyses, we used only miR-615-3p and miR-185-5p. These two miRNAs measured by NGS, yielded an AUC of 0.86 for naïve Bayes, 0.93 for logistic regression, and 0.88 for random forest (average 0.89 ± 0.04) (see Additional file 1: Table S13 for more details).

In regard to the SERS profiling, the spectral difference between the luminal and basal subtypes exhibited a complex pattern, involving several SERS bands attributed to purine metabolites and creatinine (Fig. 2D and Additional file 1: Table S8). To explore the data, PCA was performed, yielding only one PC that exhibited a statistically significant difference between the luminal and basal subtypes (PC8) (Student t-test, p < 0.001) (Fig. 2E). The relationship between the number of PCs and the explained variance is shown in Additional file 1: Fig. S5F. The loading plot of PC8 is shown in Fig. 2F. Next, the previously selected PC8 was employed as input for supervised machine learning algorithms, yielding an AUC of 0.91, 0.98, and 0.88 for naïve Bayes, logistic regression, and random forest, respectively (average 0.92 ± 0.05) (Additional file 1: Table S13).

Finally, synergisms between miRNA and SERS were found in all three classification algorithms in terms of AUC (Fig. 2G–I). Thus, the combination of miRNA and SERS profiling averaged an AUC of 0.95 ± 0.03 across the three machine learning algorithms (AUC of 0.95, 0.98, and 0.93 for naïve Bayes, logistic regression, and random forest, respectively), more than miRNA (AUC = 0.89 ± 0.04) or SERS (AUC = 0.92 ± 0.05) alone. Of note, in terms of classification accuracy, SERS alone performed better than miRNA alone or miRNA and SERS combined (Additional file 1: Table S13). These data clearly confirm that combining two liquid biopsy methods improves the diagnosis and stratification of BC.

## Discussion

Our study is the first to evaluate the combination of SERS and miRNA profiling as a diagnostic and molecular stratification tool for BC, proving the synergism between these two orthogonal liquid biopsy strategies.

We initially focused on differentiating BC patients from CTRL by identifying altered expression levels of 33 miRNAs. A panel consisting of the top three differentially expressed miRNAs (miR-34a-5p, miR-205-5p, and miR-210-3p) achieved a mean AUC of 0.84 ± 0.03 in differentiating the two groups, a result which is in line with previous reports (Braicu et al. 2019). miR-34a-5p is a tumor suppressor miRNA that inhibits BC cell motility through matrix metalloproteinase‑2 silencing (Chou et al. 2021) and is involved in cell junction regulation and epithelial to mesenchymal transition (EMT) (Braicu et al. 2019). EMT also seems to be linked with the function of miR-205-5p (Braicu et al. 2019), while miR-210-3p seems to inhibit the tumor growth and metastasis of BC via targeting fibroblast growth factor receptor-like 1 (Yang et al. 2017) and is also strongly associated with markers of tumor hypoxia like HIF-1α, CA9, Glut-1 protein (Irlam-Jones et al. 2016).

Next, we complemented miRNA profiling of urine with that of SERS. Spectral differences between BC and CTRL groups were prominent in SERS bands attributed to purine metabolites and creatinine, yielding an AUC of 0.84 ± 0.05, a result which is in line with previous reports (Li et al. 2015; Chen et al. 2019; Cui 2020; Hu 2021; Huttanus 2020). However, the present study went further in proving the clinical translatability of SERS in at least two ways. First, the SERS spectra of urine were acquired with a portable Raman spectroscope operating in a real-life clinical situation, whereas previous studies employed state-of-the-art Raman spectroscopes operated under a controlled environment. Second, the control subjects in this study were represented by patients with hematuria, a situation that better mimics the scenario encountered in the actual clinical setting, whereas previous investigations considered healthy volunteers or patients with other types of malignancies as controls. When combining miRNA and SERS data, the resulting average classification accuracy (AUC of 0.92 ± 0.06) was superior to both miRNA (AUC of 0.84 ± 0.03) and SERS alone (AUC of 0.84 ± 0.05), suggesting that the two liquid biopsy methods exhibit synergism in the diagnosis of BC. From a biological standpoint, these data show that miRNA and SERS profiling provide different information concerning the molecular status of the bladder mucosa, and these can be used for developing synergistic diagnosis tools.

In the second part of the study concerned with the molecular stratification of BC, we identified 25 miRNAs differentially expressed between the luminal and basal BC groups. Based on the top most differentially expressed miRNAs which were also confirmed by RT-qPCR (namely miR-615-3p, and miR-185-5p), the luminal and basal groups could be differentiated with an average AUC of 0.89 ± 0.04. In previous studies, urinary miR-615-3p has been shown to be overexpressed in BC patients compared to CTRLs (Wani et al. 2017), while miR-185-5p appears to target part of the inflammasome pathway, a large complex containing NOD-like receptors, that drives tumor growth and progression (Mearini et al. 2017).

When complementing miRNA profiling with SERS for better differentiation between luminal and basal groups, a synergism between the two liquid biopsy methods was also seen. Thus, the combination of miRNA and SERS profiling averaged an AUC of 0.95 ± 0.03 across the three machine learning algorithms, superior to miRNA (AUC = 0.89 ± 0.04) or SERS (AUC = 0.92 ± 0.05) alone.

Interestingly, neither miRNA nor SERS profiling could discriminate between low- and high-grade BC, suggesting that tumor grade may not be enrooted in distinct molecular features by such approaches. Further studies exploring this matter using larger cohorts are warranted.

Despite having similar pathology and clinical presentation, NMIBC tends to have different recurrence and progression rates, suggesting that the currently used diagnostic and risk stratification tools are not precise enough for a personalized follow-up and treatment, urging the development of a new molecular classification of BC as well as non-invasive diagnostic and follow-up tools (Lindskrog et al. 2021). New data suggest that the molecular fingerprint of NMIBC and MIBC better predicts the risk of recurrence, progression, and response to chemotherapy but mass implementation is difficult since complex immunohistochemical and molecular tests are required (Lindskrog et al. 2021; Audenet et al. 2018; Sjodahl 2021; Lu et al. 2021). In this context, to our knowledge, this is the first report of a liquid biopsy strategy that successfully differentiates luminal and basal BC, proving that there is a potential for a fast and accurate molecular diagnosis of BC. Further studies are necessary to establish the exact clinical relevance of molecular classification of BC and its relation to miRNA and SERS liquid biopsy strategy. The most important limitation of this study is the small sample size. To overcome this, we used three different algorithms to show that regardless of the type of classification method used, the performance of SERS combined with miRNA was higher than using any of the techniques alone. Random forest algorithm was employed due to its low sensibility to the preprocessing steps (Gromski et al. 2015). However, random forest (a non-linear algorithm) yielded the lowest classification accuracy based on SERS, miRNA, and the combination of SERS and miRNA data. We suppose we obtained these results mainly because of its higher sensibility to a low number of input data compared to linear algorithms. To overcome the limitation of the low number of samples, we used logistic regression and naïve Bayes for the classification of BC and CTRL, low- and high-grade, and basal and luminal BC based on SERS and miRNA data. Even if the logistic regression model is the most widely used statistical technique nowadays for binary medical outcomes (Steyerberg 2009), the naïve Bayes classifier was associated with the highest performances in the classification of BC and CTRL group based on miRNA only, SERS only, and miRNA and SERS together. The minimal user implication for the naïve Bayes algorithm recommends it for the standardization of analysis such as SERS liquid biopsy.

## Conclusions

In this study, we demonstrated that urine miRNA profiling synergizes with SERS profiling for a better BC's diagnostic and molecular stratification. We consider that by combining two liquid biopsy methods, a clinically relevant tool that can aid BC patients can be developed.

## Supplementary Information

**Additional file 1: Figure S1. **Differentially expressed miRNAs in urine of patients with bladder cancer (BC) and controls (CTRL) (prospective cohort). **A** Volcano plot showing the differentially expressed urine miRNAs between BC patients and CTRLs in the NGS analysis. The color intensity of the dots represents the expression level (as the log_10_ of the normalized mean counts), the x-axis represents the fold change, and the y-axis the nominal p-value. **B** Dot plot showing the statistical significance of the functional terms identified as enriched in the target genes of differentially expressed miRNAs between BC cases and controls. The size of the dot is proportional to the number of target genes belonging to each term, while the color code refers to the coefficient computed by RBiomirGS. Negative (in blue) and positive (in red) coefficients represent processes predicted to be, respectively, downregulated or upregulated based on the miRNA expression change between the two groups. **C** The expression of the three candidate miRNAs analyzed by RT-qPCR. The expression was normalized with the mean of 7 miRNAs used for this study. Mean ± SD. *p < 0.05, ***p < 0.001. **Figure S2. **Validation of the differentially expressed candidate miRNAs in urine of patients with bladder cancer (BC, n = 66) and controls (CTRL, n = 50) (retrospective cohort). The expression of the three candidate miRNAs (miR-34a-5p, miR-205-5p and miR-210-3p) analyzed by NGS. Mean ± SD. ***p < 0.001, ****p < 0.0001. **Figure S3. **SERS profiling for classifying bladder cancer (BC) and controls (CTRL).** A** Loading plots of the principal components that showed no statistical relevance in the classification of BC and CTRLs based on SERS spectra of urine samples. **B** The relationship between the number of principal components (x-axis) and the explained variance in the original dataset (y-axis) of BC cases versus CTRLs. The first 11 principal components explain 98.3% of the variance in the original dataset. **Figure S4. **Urine liquid biopsy by combining miRNA and SERS profiling for classifying low- and high-grade bladder cancer (BC). **A** Representative H&E staining of low-grade (LG) versus high-grade (HG) BC patients. **B** Volcano plot of differentially expressed miRNAs by NGS analysis in the urine of LG and HG BC patients. The color intensity of the dots represents the expression level, the x-axis represents the fold change, and the y-axis the nominal p-value. **C.** Heat map of the differentially expressed miRNAs in urine between LG and HG BC patients. The color scale shows the log_10_ of the normalized counts. **D** Normalized expression levels of miR-1246 for LG and HG BC measured by NGS. **E** Relative expression levels of miR-1246 in LG and HG BC measured by RT-qPCR. **F** Normalized expression levels of miR-1246 for LG and HG BC of the miRNA validation cohort measured by NGS. **G** The average SERS spectrum of urine from LG versus HG BC patients. **H** The distribution of score values for principal component (PC) PC5 and PC6 of patients with LG (red) versus HG BC (blue). **I** Receiver operating characteristic (ROC) curve for the classification of LG and HG BC achieved by naïve Bayes algorithm run on datasets consisting of the differentially expressed miRNA alone (miR-1246), SERS data alone (first 11 PCs), or a combination of the two. **J** ROC curve for the classification of LG and HG BC achieved by logistic regression algorithm run on datasets consisting of the differentially expressed miRNA alone (miR-1246), SERS data alone (first 11 PCs), or a combination of the two. **K** The ROC curve for the classification of LG and HG BC achieved by random forest algorithm run on datasets consisting of the differently expressed miRNA alone (miR-1246), SERS data alone (first 11 PCs), or a combination of the two. Mean ± SD. ns = not significant, **p < 0.01. **Figure S5. **Urine liquid biopsy by combining miRNA and SERS profiling for classifying luminal and basal type bladder cancer (BC). **A** Representative immunohistochemistry (IHC) staining for TP53 mutated samples (TP53 accumulating nuclear), diffuse nuclear staining for GATA3 (100% of the tumor cells), weak ER staining (17.5%), and complete, circumferential staining of cell membranes for Her2neu (100% of the tumor cells = score 3). **B** Volcano plot of differentially expressed miRNAs by NGS analysis in the urine of luminal and basal BC patients. The color intensity of the dots represents the expression levels, the x-axis represents the fold change, and the y-axis the nominal p-value. **C** Heat map of the differentially expressed miRNAs by NGS analysis in urine between luminal and basal BC. The color scale shows the log_10_ of the normalized counts. **D** Normalized expression levels of miR-204-5p in luminal and basal type BC measured by NGS. **E** Relative expression levels of miR-615-3p, miR-185-5p, and miR-204-5p in luminal and basal type BC measured by RT-qPCR. The expression was normalized with the mean of 7 miRNAs used for this study. **F** The relationship between the number of principal components (x-axis) and the explained variance in the original dataset (y-axis) of luminal versus basal BC. The first 11 principal components explain around 98% of the variance in the original dataset. Mean ± SD. *p < 0.05, **p < 0.01, ****p < 0.0001. **Table S1.** Demographic data and information concerning patients with bladder cancer (BC) (grade, TNM, muscle invasiveness, evolution at 3 months) and controls (CTRL). **Table S2.** Demographic data and information concerning patients with bladder cancer (BC) (grade, T stage, muscle invasiveness, recurrence) and controls from the retrospective validation cohort. **Table S3**. Table with a detailed description of the antibodies used in this study. **Table S4**. Detailed presentation of the Allred score for TP53 analysis. **Table S5.** Differentially expressed miRNAs in urine between bladder cancer patients and control subjects. **Table S7**. The diagnostic ability to distinguish bladder cancer and control group patients with three classification algorithms (naïve Bayes, logistic regression, and random forest) run on datasets consisting of the top three differently expressed miRNAs alone (miR-34a-5p, miR-205-5p, and miR-210-3p), SERS data alone, or a combination of the two (miRNA + SERS). **Table S8.** Tentative assignment of the SERS bands based. **Table S9**. Differentially expressed miRNA between the urine of low-grade and high-grade bladder cancer patients. **Table S10**. The diagnostic ability to distinguish low- and high-grade bladder cancer with three classification algorithms (naïve Bayes, logistic regression, and random forest) run on datasets consisting of the only differentially expressed miRNA (miR-1246), SERS data alone (first 11 principal components (PC)), or a combination of the two (miRNA + SERS). **Table S11**. Immunohistochemistry-based analysis of the included BC patients. **Table S12.** Differentially expressed urinary miRNAs between luminal and basal type bladder cancer patients. **Table S13**. The diagnostic ability to distinguish the luminal type and basal type bladder cancer with the three classification algorithms (naïve Bayes, logistic regression, and random forest) run on datasets consisting of top three differentially expressed miRNAs alone (miR-204-5p, miR-615-3p, and miR-185-5p), SERS data alone, or a combination of the two (miRNA + SERS).

**Additional file 2: Table S6. A** The list of gene sets significantly enriched in targets of miRNAs downregulated or upregulated in the differential expression analysis between cases and controls. **B** List of the validated miRNA-target interactions used for the enrichment analysis (Table attached as an additional Excel File).

## Data Availability

The data that support the findings of this study are available from the corresponding authors upon reasonable request.

## Abbreviations

AUC: Area under the curve
BC: Bladder cancer
CTRL: Controls
EMT: Epithelial to mesenchymal transition
LOO: Leave-one-out
miRNA: MicroRNA
NGS: Next generation sequencing
PC: Principal component
PCA: Principal component analysis
ROC: Receiver operating characteristic
sncRNA: Small non-coding RNA
SERS: Aurface-enhanced raman spectroscopy
