# Supplementary material for: Combined miRNA and SERS urine liquid biopsy for the point-of-care diagnosis and molecular stratification of bladder cancer
Source: Mol Med. 2022 Apr 1;28:39. doi: 10.1186/s10020-022-00462-z (PMC8973824; doi:10.1186/s10020-022-00462-z)
Supplement: Supplementary file 1 — Additional file 1: Figure S1. Differentially expressed miRNAs in urine of patients with bladder cancer (BC) and controls (CTRL) (prospective cohort). A Volcano plot showing the differentially expressed urine miRNAs between BC patients and CTRLs in the NGS analysis. The color intensity of the dots represents the expression level (as the log10 of the normalized mean counts), the x-axis represents the fold change, and the y-axis the nominal p-value. B Dot plot showing the statistical significance of the functional terms identified as enriched in the target genes of differentially expressed miRNAs between BC cases and controls. The size of the dot is proportional to the number of target genes belonging to each term, while the color code refers to the coefficient computed by RBiomirGS. Negative (in blue) and positive (in red) coefficients represent processes predicted to be, respectively, downregulated or upregulated based on the miRNA expression change between the two groups. C The expression of the three candidate miRNAs analyzed by RT-qPCR. The expression was normalized with the mean of 7 miRNAs used for this study. Mean ± SD. *p < 0.05, ***p < 0.001. Figure S2. Validation of the differentially expressed candidate miRNAs in urine of patients with bladder cancer (BC, n = 66) and controls (CTRL, n = 50) (retrospective cohort). The expression of the three candidate miRNAs (miR-34a-5p, miR-205-5p and miR-210-3p) analyzed by NGS. Mean ± SD. ***p < 0.001, ****p < 0.0001. Figure S3. SERS profiling for classifying bladder cancer (BC) and controls (CTRL). A Loading plots of the principal components that showed no statistical relevance in the classification of BC and CTRLs based on SERS spectra of urine samples. B The relationship between the number of principal components (x-axis) and the explained variance in the original dataset (y-axis) of BC cases versus CTRLs. The first 11 principal components explain 98.3% of the variance in the original dataset. Figure S4. Urine liquid [file 10020_2022_462_MOESM1_ESM.docx]

**Combined miRNA and SERS urine liquid biopsy for the point-of-care diagnosis and molecular stratification of bladder cancer**

Tudor Moisoiu^1,2,3,†^, Mihnea P. Dragomir^4,5,†,*^, Stefania D. Iancu^6,†^, Simon Schallenberg^4^, Giovanni Birolo^7^, Giulio Ferrero^8^, Dan Burghelea^1,2^, Andrei Stefancu^6^, Ramona G. Cozan^6^, Emilia Licarete^9^, Alessandra Allione^7^, Giuseppe Matullo^7^, Gheorghita Iacob^1^, Zoltán Bálint^6^,

Radu I. Badea^2,10^, Alessio Naccarati^11,12^, David Horst^4,5^, Barbara Pardini^11,12,*^,

Nicolae Leopold^3,6,*^, Florin Elec^1,2,*^

^1^Clinical Institute of Urology and Renal Transplantation, 400006 Cluj-Napoca, Romania

^2^Iuliu Hatieganu University of Medicine and Pharmacy, 400012 Cluj-Napoca, Romania

^3^Biomed Data Analytics SRL, 400696 Cluj-Napoca, Romania

^4^Institute of Pathology, Charité-Universitätsmedizin Berlin, corporate member of Freie Universität Berlin, Humboldt-Universität zu Berlin and Berlin Institute of Health, 10117 Berlin, Germany

^5^German Cancer Consortium (DKTK), Partner Site Berlin, and German Cancer Research Center (DKFZ), Heidelberg, Germany

^6^Faculty of Physics, Babeș-Bolyai University, 400084 Cluj-Napoca, Romania

^7^Department of Medical Sciences, University of Turin, 10126 Turin, Italy

^8^Department of Clinical and Biological Sciences, University of Turin, Regione Gonzole, 10, 10043 Orbassano, Italy

^9^Faculty of Biology, Babeș-Bolyai University, 400015 Cluj-Napoca, Romania

^10^Octavian Fodor Regional Institute of Gastroenterology and Hepatology, 400162 Cluj-Napoca, Romania

^11^Candiolo Cancer Institute-FPO IRCCS, 10060 Candiolo, Turin, Italy

^12^Italian Institute for Genomic Medicine (IIGM), c/o IRCCS Candiolo, 10060 Candiolo, Turin, Italy

^†^Authors with equal contribution

^*^**Corresponding authors**: mihnea.dragomir@charite.de; barbara.pardini@iigm.it; nicolae.leopold@ubbcluj.ro, and ioan.elec@umfcluj.ro.

# **Supplementary Figures**

**
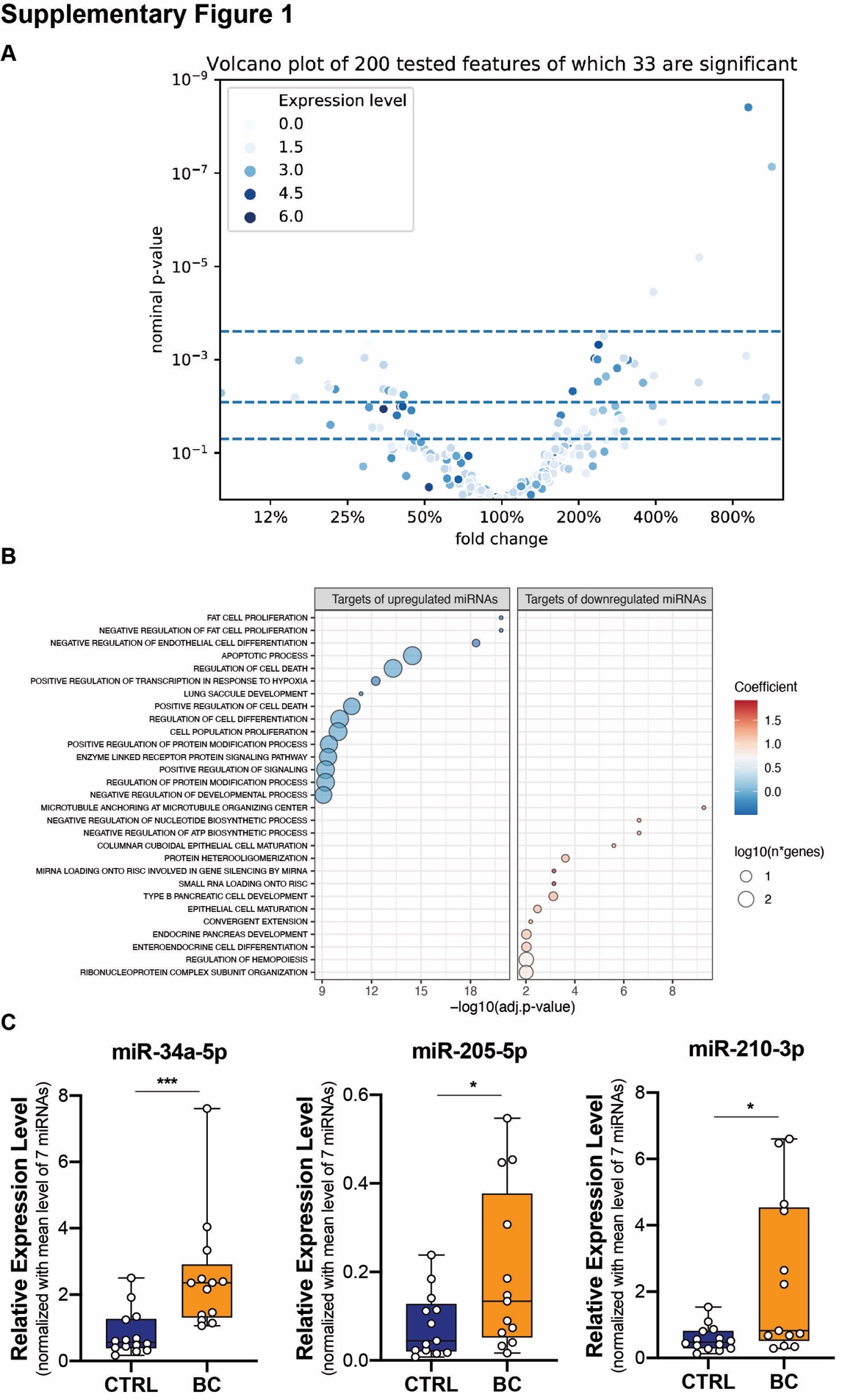
**

**Supplementary Figure 1.** **Differentially expressed** **miRNAs in urine of patients with bladder cancer and controls (prospective cohort).**

**A**. Volcano plot showing the differentially expressed urine miRNAs between BC patients and CTRLs in the NGS analysis. The color intensity of the dots represents the expression level (as the log_10_ of the normalized mean counts), the x-axis represents the fold change and the y-axis the nominal p-value. **B**. Dot plot showing the statistical significance of the functional terms identified as enriched in the target genes of differentially expressed miRNAs between BC cases and controls. The size of the dot is proportional to the number of target genes belonging to each term, while the color code refers to the coefficient computed by RBiomirGS. Negative (in blue) and positive (in red) coefficients represent processes predicted to be, respectively, downregulated or upregulated based on the miRNA expression change between the two groups. **C**. The expression of the three candidate miRNAs analyzed by RT-qPCR. The expression was normalized with the mean of 7 miRNAs used for this study. Mean ± SD. *p < 0.05, ***p < 0.001.


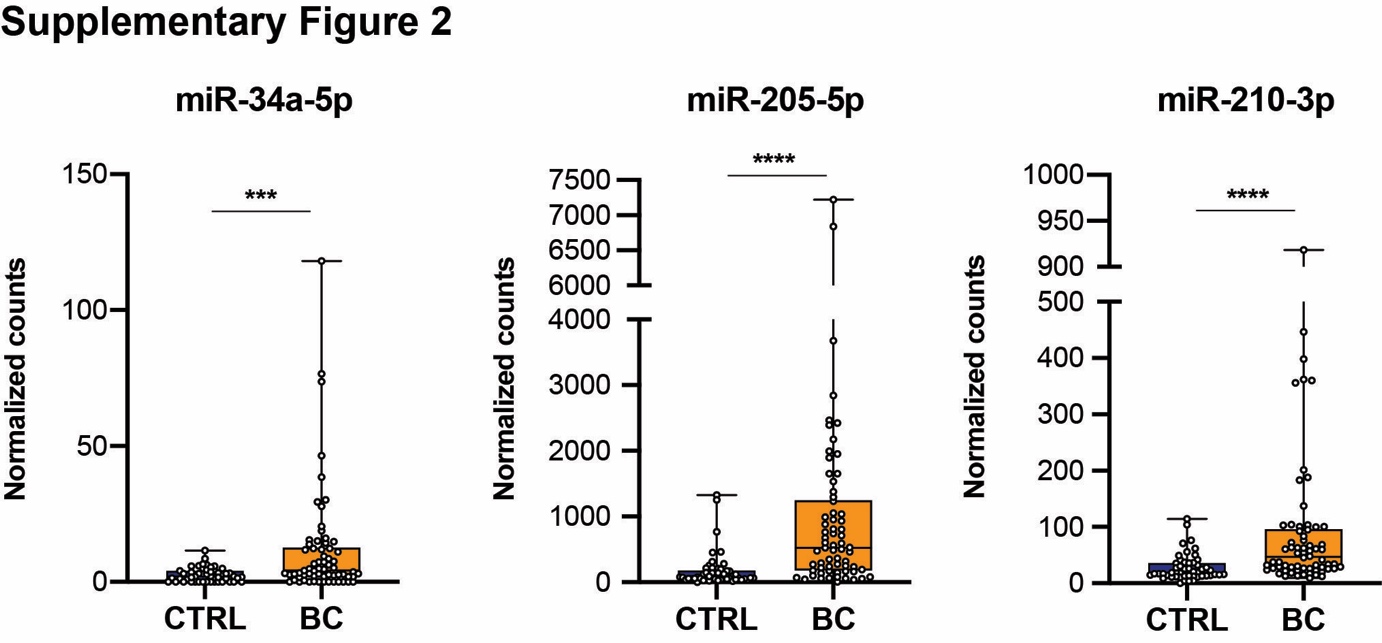


**Supplementary Figure 2.** **Validating the differentially expressed** **candidate** **miRNAs in urine of patients with bladder cancer (n = 66) and controls (n = 50) (retrospective cohort).**

The expression of the three candidate miRNAs (miR-34a-5p, miR-205-5p and miR-210-3p) analyzed by NGS. Mean ± SD. ***p < 0.001, ****p < 0.0001.

**
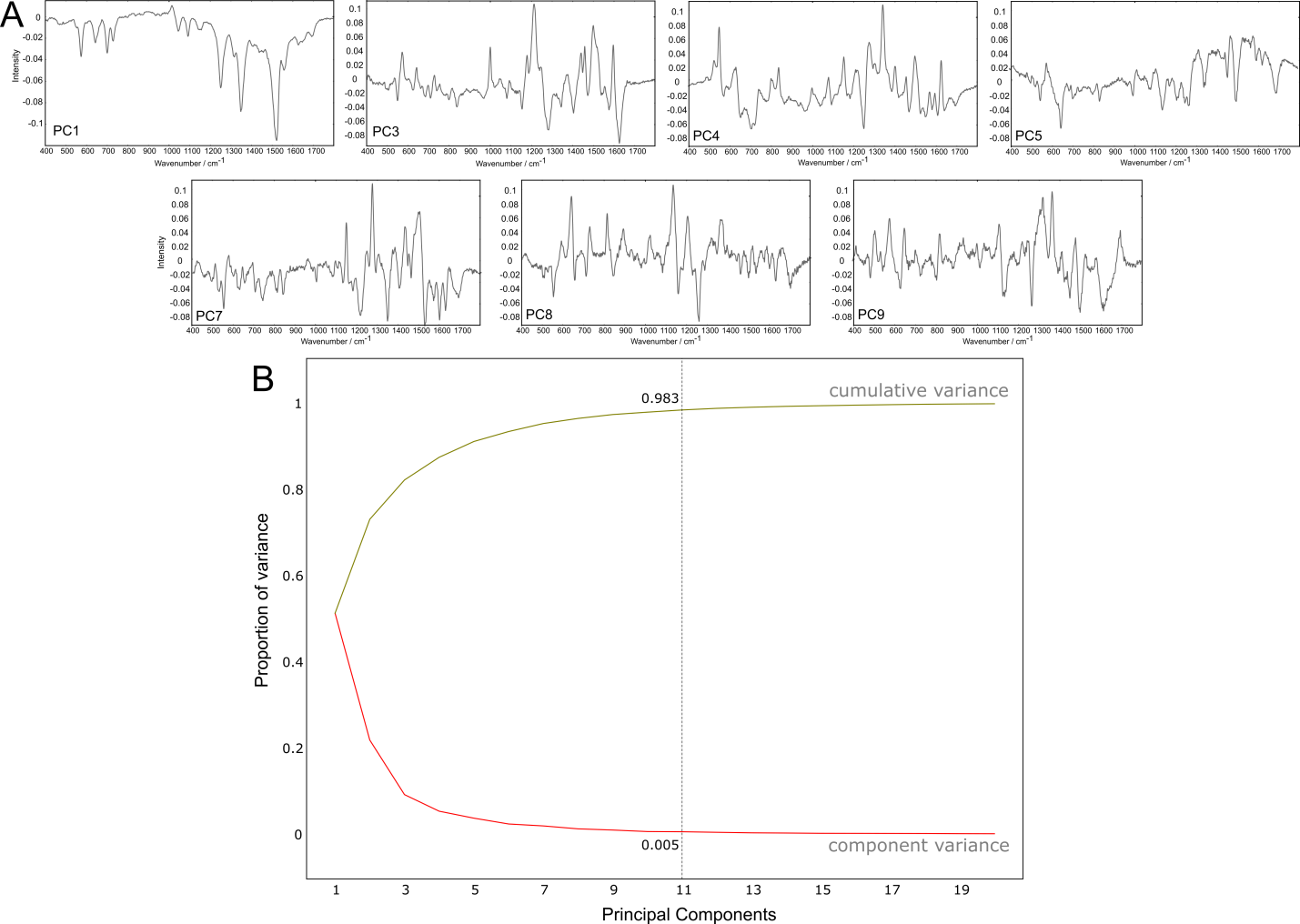
**

**Supplementary Figure 3.** **SERS profiling for classifying bladder cancer and controls.**

**A**. Loadings plot of the principal components that showed no statistical relevance in the classification of bladder cancer (BC) and controls (CTRLs) based on SERS spectra of urine samples. **B**. The relationship between the number of principal components (x-axis) and the explained variance in the original dataset (y-axis) of BC cases versus CTRLs. The first 11 principal components explain 98.3% of the variance in the original dataset.

**
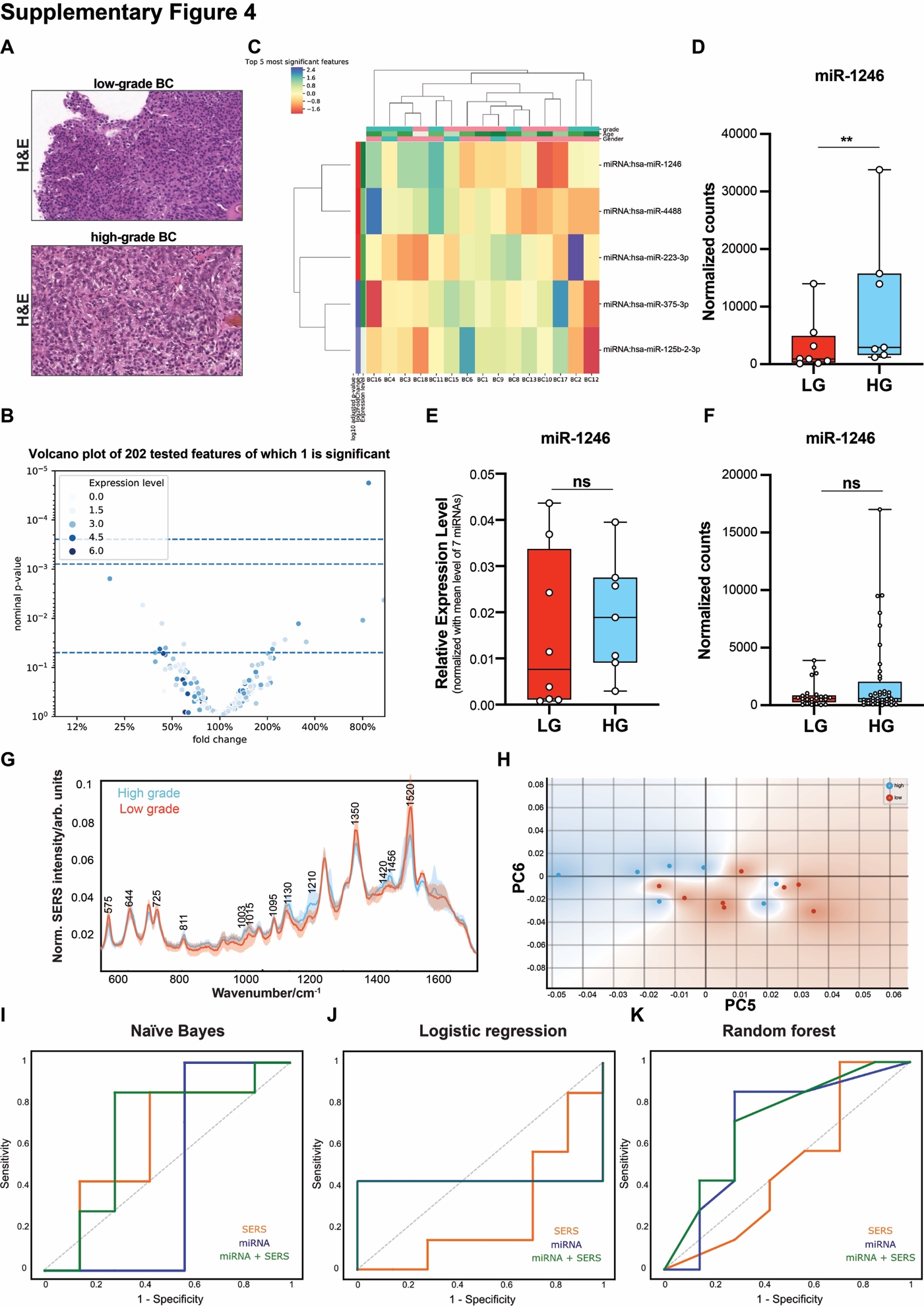
**

**Supplementary Figure 4. Urine liquid biopsy by combining miRNA and SERS profiling for classifying low- and high-grade bladder cancer (BC).**

**A.** Representative H&E staining of low-grade (LG) versus high-grade (HG) BC patients. **B.** Volcano plot of differentially expressed miRNAs by NGS analysis in the urine of low- and high-grade BC patients. The color intensity of the dots represents the expression level, the x-axis represents the fold change, and the y-axis the nominal p-value. **C.** Heat map of the differentially expressed miRNAs in urine between low-grade and high-grade BC patients. The color scale shows the log10 of the normalized counts. **D.** Normalized expression levels of miR-1246 for low- and high-grade BC measured by NGS. **E.** Relative expression levels of miR-1246 in low- and high-grade BC measured by RT-qPCR. **F.** Normalized expression levels of miR-1246 for low- and high-grade BC of the miRNA validation cohort measured by NGS. **G.** The average SERS spectrum of urine from low-grade versus high-grade BC patients. **H.** The distribution of score values for principal component (PC) PC5 and PC6 of patients with low-grade (red) versus high-grade BC (blue). **I.** Receiver operating characteristic (ROC) curve for the classification of low-grade and high-grade BC achieved by naïve Bayes algorithm run on datasets consisting of differentially expressed miRNAs alone (miR-1246), SERS data alone (first 11 PCs), or a combination of the two. **J.** ROC curve for the classification of low-grade and high-grade BC achieved by logistic regression algorithm run on datasets consisting of differently expressed miRNAs alone (miR-1246), SERS data alone (first 11 PCs), or a combination of the two. **K.** The ROC curve for the classification of low-grade and high-grade BC achieved by random forest algorithm run on datasets consisting of differently expressed miRNAs alone (miR-1246), SERS data alone (first 11 PCs), or a combination of the two. Mean ± SD. ns = not significant, **p < 0.01.

**
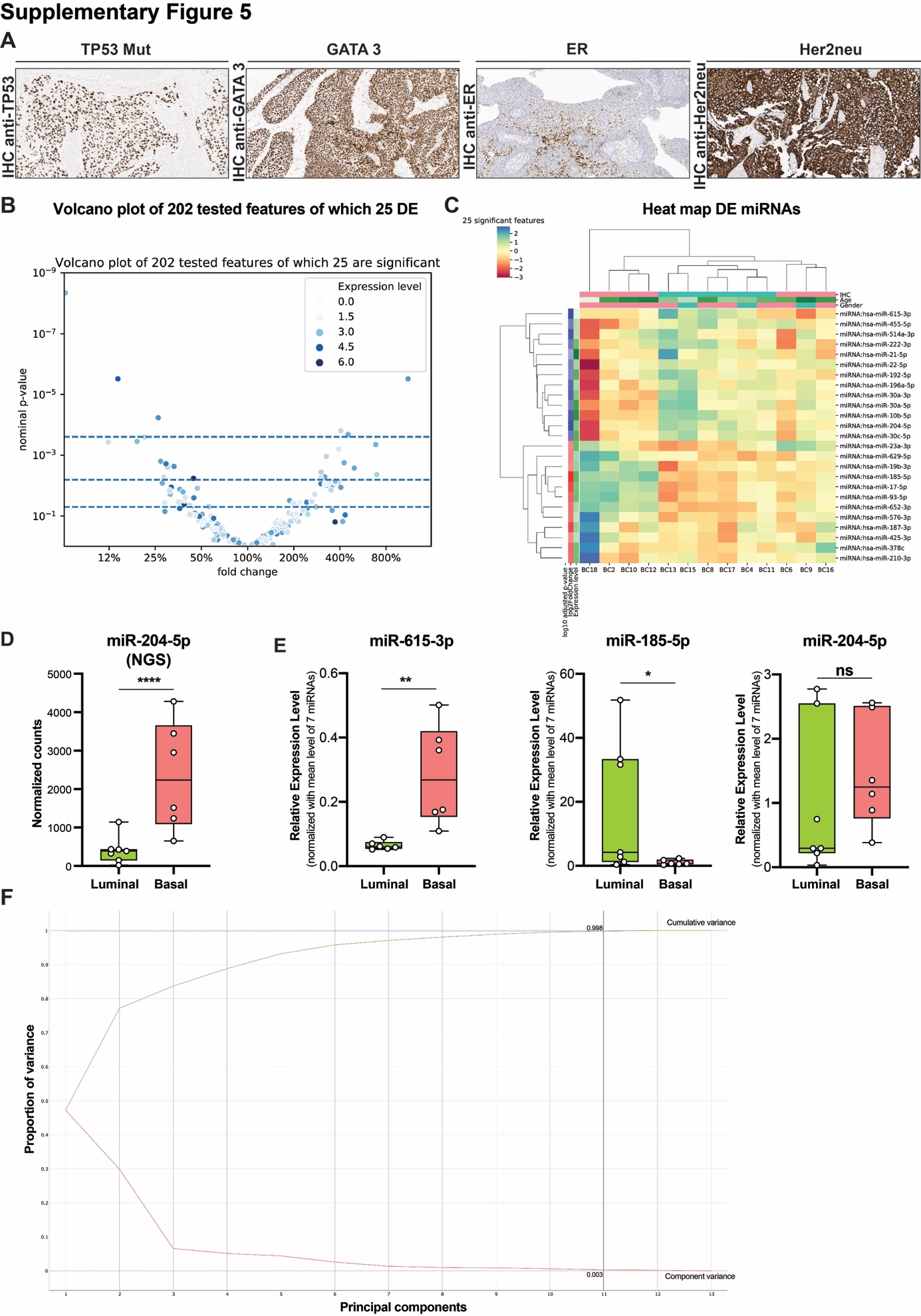
**

**Supplementary Figure 5.** **Urine liquid biopsy by combining miRNA and SERS profiling for classifying luminal type and basal type bladder cancer (BC)**.

**A.** Representative immunohistochemistry (IHC) staining for TP53 mutated samples (TP53 accumulating nuclear), diffuse nuclear staining for GATA3 (100% of the tumor cells), weak ER staining (17.5%), and complete, circumferential staining of cell membranes for HER2neu (100% of the tumor cells = score 3). **B.** Volcano plot of differentially expressed miRNAs by NGS analysis in urine of luminal and basal BC patients. The color intensity of the dots represents the expression levels, the x-axis represents the fold change and the y-axis the nominal p-value. **C.** Heat map of the differentially expressed miRNAs by NGS analysis in urine between luminal and basal BC. The color scale shows the log_10_ of the normalized counts. **D.** Normalized expression levels of miR-204-5p in luminal and basal type BC measured by NGS. **E**. Relative expression levels of miR-615-3p, miR-185-5p, and miR-204-5p in luminal and basal type BC measured by RT-qPCR. The expression was normalized with the mean of 7 miRNAs used for this study. **E**. The relationship between the number of principal components (x-axis) and the explained variance in the original dataset (y-axis) of luminal versus basal BC. The first 11 principal components explain around 98% of the variance in the original dataset. Mean ± SD. *p < 0.05, **p < 0.01, ****p < 0.0001.

# **Supplementary Tables**

**Supplementary Table S1.** Demographic data and information concerning patients with bladder cancer (BC) (grade, TNM, muscle invasiveness, evolution at 3 months) and controls (CTRL).

| Category | Name | Sex | Grade | Stage | NMIBC/MIBC | Evolution at 3 months |
| --- | --- | --- | --- | --- | --- | --- |
| BC | BC1 | M | low | TaN0M0 | NMIBC | No recurrence |
| BC | BC2 | M | high | T3aN1M0 | MIBC | Radical cystectomy |
| BC | BC3 | M | high | T1N0M0 | NMIBC | No recurrence |
| BC | BC4 | F | high | TaN0M0 | NMIBC | Recurrence |
| BC | BC5 | M | low | TaN0M0 | NMIBC | No recurrence |
| BC | BC6 | M | high | T2N0M0 | MIBC | No recurrence |
| BC | BC7 | F | low | T1N0M0 | NMIBC | No recurrence |
| BC | BC8 | M | low | TaN0M0 | NMIBC | No recurrence |
| BC | BC9 | M | high | T2N0M0 | MIBC | Radical cystectomy |
| BC | BC10 | M | high | T1N0M0 | NMIBC | No follow-up |
| BC | BC11 | M | low | TaN0M0 | NMIBC | No follow-up |
| BC | BC12 | F | low | TaN0M0 | NMIBC | No recurrence |
| BC | BC13 | M | high | TaN0M0 | NMIBC | No follow-up |
| BC | BC14 | M | low | TaN0M0 | NMIBC | No recurrence |
| BC | BC15 | M | low | TaN0M0 | NMIBC | Radical cystectomy |
| CTRL | C1 | F |  |  |  |  |
| CTRL | C2 | F |  |  |  |  |
| CTRL | C3 | F |  |  |  |  |
| CTRL | C4 | M |  |  |  |  |
| CTRL | C5 | M |  |  |  |  |
| CTRL | C6 | M |  |  |  |  |
| CTRL | C7 | F |  |  |  |  |
| CTRL | C8 | M |  |  |  |  |
| CTRL | C9 | F |  |  |  |  |
| CTRL | C10 | F |  |  |  |  |
| CTRL | C11 | F |  |  |  |  |
| CTRL | C12 | F |  |  |  |  |
| CTRL | C13 | F |  |  |  |  |
| CTRL | C14 | F |  |  |  |  |
| CTRL | C15 | F |  |  |  |  |
| CTRL | C16 | F |  |  |  |  |

**Supplementary Table S2.** Demographic data and information concerning patients with bladder cancer (BC) (grade, T stage, muscle invasiveness, recurrence) and controls from the retrospective validation cohort.

| Variables | BC  (n = 66) | CTRL  (n = 50) |
| --- | --- | --- |
| Age (mean) | 64.27 | 64.64 |
| Sex | M = 66 | M = 50 |
| Grade | LG = 25, HG = 41 | - |
| Tumor stage | Tis = 4, Ta = 29,  T1 = 24, ≥T2 = 10 | - |
| NMIBC/MIBC | NMIBC = 56, MIBC = 10 | - |
| Recurrence | Yes = 25, No = 41 | - |
| Progression | Yes = 2, No = 64 | - |
| Status | Alive = 58, Dead = 8 | - |

**Supplementary Table S3**. Table with detailed description of the antibodies used in this study.

| **Antigen** | **Abbreviation** | **Clone** | **Manufacturer** | **Catalog #** | **Dilution** |
| --- | --- | --- | --- | --- | --- |
| Tumor suppressor protein 53 | p53 | DO-7 | Dako | M7001 | 1:50 |
| Estrogen receptor alpha | ER | SP1 | Ventana | 790-4324 | Ready to use |
| GATA Transcription factor 3 | GATA 3 | EP368 | Epitomics | AC-0324 | 1:50 |
| Her2/neu | Her2neu | 4B5 | Ventana | 790-4493 | Ready to use |
| Keratin 5/6 | CK 5/6 | EP24, EP67 | Abcam | AC-9001RUOBUL | 1:100 |
| CD44 Protein | CD 44 | DF1485 | Dako | M7082 | 1:50 |
| Keratin 20 | CK 20 | KS20.8 | Dako | M7019 | 1:100 |
| Uroplakin III | UP III | AU1 | Progen | 651108 | Ready to use |

**Supplementary Table S4.** Detailed presentation of the Allred score for P53 analysis.

| Proportion score | |
| --- | --- |
| Percentage of stained tumor cell nuclei | Equivalent |
| 0% | 0 |
| > 0-1% | 1 |
| > 1-10% | 2 |
| > 10-33% | 3 |
| > 33-66% | 4 |
| > 66-100% | 5 |
| Intensity score | |
| Intensity of nuclear staining | Equivalent |
| Negative | 0 |
| Low | 1 |
| Moderate | 2 |
| High | 3 |

**Supplementary Table S5.** Differentially expressed miRNAs in urine between bladder cancer patients and control subjects.

| MiRNA | Base mean | Differential expression | log2 Fold Change | Adjusted p value | |
| --- | --- | --- | --- | --- | --- |
| hsa-miR-205-5p | 4107.255 | Upregulated | 3.201464 | | 7.79E-07 |
| hsa-miR-210-3p | 522.2282 | Upregulated | 3.505753 | | 7.34E-06 |
| hsa-miR-34a-5p | 43.99834 | Upregulated | 2.563589 | | 0.000428 |
| hsa-miR-574-3p | 51.57244 | Upregulated | 1.968494 | | 0.001771 |
| hsa-miR-342-3p | 62.26486 | Upregulated | 1.326932 | | 0.012646 |
| hsa-miR-200b-3p | 21781.49 | Upregulated | 1.25727 | | 0.013714 |
| hsa-miR-628-3p | 18.65372 | Downregulated | -1.73003 | | 0.013714 |
| hsa-miR-514a-3p | 306.737 | Downregulated | -2.63006 | | 0.014826 |
| hsa-miR-429 | 3372.517 | Upregulated | 1.242284 | | 0.014826 |
| hsa-miR-424-3p | 117.8028 | Downregulated | -1.78216 | | 0.014826 |
| hsa-miR-934 | 128.9483 | Upregulated | 1.584635 | | 0.014826 |
| hsa-miR-378a-3p | 3979.694 | Upregulated | 1.638348 | | 0.014826 |
| hsa-miR-200c-3p | 22612.06 | Upregulated | 1.205655 | | 0.014826 |
| hsa-miR-4497 | 49.30248 | Upregulated | 3.1744 | | 0.014826 |
| hsa-miR-19b-3p | 148.9219 | Upregulated | 1.721376 | | 0.016205 |
| hsa-let-7d-3p | 189.092 | Downregulated | -1.53298 | | 0.016205 |
| hsa-miR-320a-3p | 3111.669 | Upregulated | 1.498542 | | 0.017994 |
| hsa-miR-769-5p | 51.59213 | Upregulated | 1.973653 | | 0.024252 |
| hsa-miR-148b-3p | 656.7228 | Upregulated | 1.353086 | | 0.024252 |
| hsa-miR-187-3p | 127.5943 | Upregulated | 2.555957 | | 0.028568 |
| hsa-miR-98-5p | 1272.776 | Upregulated | 1.248984 | | 0.028568 |
| hsa-miR-23a-3p | 892.6995 | Upregulated | 1.834158 | | 0.028568 |
| hsa-miR-142-3p | 114.6504 | Downregulated | -2.25485 | | 0.030353 |
| hsa-miR-9985 | 52.27248 | Downregulated | -2.24439 | | 0.032862 |
| hsa-miR-204-5p | 2533.559 | Downregulated | -2.1622 | | 0.033283 |
| hsa-miR-10b-3p | 166.052 | Downregulated | -1.53631 | | 0.033283 |
| hsa-miR-194-5p | 1235.804 | Downregulated | -1.47024 | | 0.033699 |
| hsa-miR-200a-3p | 10449.55 | Upregulated | 0.921323 | | 0.033699 |
| hsa-miR-501-3p | 71.19647 | Downregulated | -1.41166 | | 0.033699 |
| hsa-miR-223-5p | 510.4043 | Downregulated | -3.64252 | | 0.034614 |
| hsa-miR-128-3p | 1214.365 | Downregulated | -1.2717 | | 0.036669 |
| hsa-miR-615-3p | 48.42762 | Downregulated | -2.68389 | | 0.039309 |
| hsa-miR-4488 | 189.4169 | Upregulated | 3.431179 | | 0.039309 |

**Supplementary Table S6. A.** The list of gene sets significantly enriched in targets of miRNAs downregulated or upregulated in the differential expression analysis between cases and controls. **B.** List of the validated miRNA-target interactions used for the enrichment analysis (Table attached as an additional **Excel File**).

**Supplementary Table S7**. The diagnostic ability to distinguish bladder cancer and control group patients with three classification algorithms (naïve Bayes, logistic regression, and random forest) run on datasets consisting of the top three differently expressed miRNAs alone (miR-34a-5p, miR-205-5p and miR-210-3p), SERS data alone, or a combination of the two (miRNA + SERS).

| Model | input | AUC | CA | F1 | Precision | Recall |
| --- | --- | --- | --- | --- | --- | --- |
| Logistic Regression | miRNA | 0.84 | 0.74 | 0.74 | 0.74 | 0.74 |
|  | SERS | 0.87 | 0.84 | 0.84 | 0.84 | 0.84 |
|  | miRNA + SERS | 0.94 | 0.90 | 0.90 | 0.90 | 0.90 |
| Naïve Bayes | miRNA | 0.87 | 0.77 | 0.77 | 0.77 | 0.77 |
|  | SERS | 0.86 | 0.84 | 0.84 | 0.84 | 0.84 |
|  | miRNA + SERS | 0.97 | 0.93 | 0.93 | 0.93 | 0.93 |
| Random Forest | miRNA | 0.81 | 0.74 | 0.74 | 0.74 | 0.74 |
|  | SERS | 0.78 | 0.71 | 0.71 | 0.71 | 0.71 |
|  | miRNA + SERS | 0.86 | 0.74 | 0.74 | 0.74 | 0.74 |

AUC = Area under the ROC curve; CA = classification accuracy; F1 = harmonic mean of precision and recall.

**Supplementary Table S8.** Tentative assignment of the SERS bands based on data from (1-5).

| SERS band (cm^-1^) | Assignment |
| --- | --- |
| 575 | Creatinine |
| 644 | Uric acid |
| 700 | Creatinine |
| 725 | Uric acid/Hypoxanthine |
| 811 | Uric acid |
| 936 | Unknown |
| 1003 | Urea |
| 1015 | Uric acid |
| 1048 | Unknown |
| 1095 | Hypoxanthine |
| 1130 | Uric acid |
| 1210 | Uric acid |
| 1250 | Unknown |
| 1350 | Uric acid |
| 1420 | Creatinine |
| 1456 | Creatinine |
| 1520 | Uric acid |
| 1562 | Unknown |
| 1598 | Unknown |
| 1685 | Uric acid |

**Supplementary Table S9**. Differentially expressed miRNA between the urine of low-grade and high-grade bladder cancer patients.

| MiRNA | Base mean | Differential expression | log2 Fold Change | Adjusted p value |
| --- | --- | --- | --- | --- |
| hsa-miR-1246 | 6485.014 | Upregulated | 3.126375 | 0.003577 |

**Supplementary Table S10**. The diagnostic ability to distinguish low-grade and high-grade bladder cancer with three classification algorithms (naïve Bayes, logistic regression, and random forest) run on datasets consisting of the only differentially expressed miRNA (miR-1246), SERS data alone (first 11 principal components (PC)), or a combination of the two (miRNA + SERS).

| Model | Input | AUC | CA | F1 | Precision | Recall |
| --- | --- | --- | --- | --- | --- | --- |
| Logistic Regression | miRNA | 0.37 | 0.60 | 0.59 | 0.60 | 0.60 |
|  | SERS (first 11 PCs) | 0.30 | 0.27 | 0.26 | 0.26 | 0.27 |
|  | miRNA + SERS | 0.39 | 0.40 | 0.39 | 0.39 | 0.40 |
| Naïve Bayes | miRNA | 0.37 | 0.53 | 0.52 | 0.55 | 0.53 |
|  | SERS (first 11 PCs) | 0.61 | 0.47 | 0.47 | 0.47 | 0.47 |
|  | miRNA + SERS | 0.64 | 0.53 | 0.53 | 0.53 | 0.53 |
| Random Forest | miRNA | 0.66 | 0.60 | 0.60 | 0.60 | 0.60 |
|  | SERS (first 11 PCs) | 0.46 | 0.40 | 0.40 | 0.40 | 0.40 |
|  | miRNA + SERS | 0.62 | 0.53 | 0.53 | 0.53 | 0.53 |

AUC = Area under the ROC curve; CA = classification accuracy; F1 = harmonic mean of precision and recall.

**Supplementary Table S11**. Immunohistochemistry-based analysis of the included BC patients.

| Sample | P53 | | | | Basal | | Luminal | | Basal/Lumin. | GATA 3 | Her2neu | ER |
| --- | --- | --- | --- | --- | --- | --- | --- | --- | --- | --- | --- | --- |
|  | **PS** | **IS** | **Total** | **WT/Mut** | **CD44** | **CK5/6** | **CK20** | **Uroplakin III** |  |  |  |  |
| BC1 | N/A | N/A | N/A | N/A | N/A | N/A | N/A | N/A | N/A | N/A | N/A | N/A |
| BC2 | 5 | 3 | 8 | **Mut** | 47.5% | 5.0% | 20.0% | 70.0% | Luminal | 100.0% | 0 | 1.0% |
| BC3 | N/A | N/A | N/A | N/A | N/A | N/A | N/A | N/A | N/A | N/A | N/A | N/A |
| BC4 | 3 | 2 | 5 | WT | 70.0% | 17.5% | 60.0% | 50.0% | Basal | 100.0% | 3 | 1.0% |
| BC5 | 4.5 | 1.5 | 6 | WT | 47.5% | 12.5% | 62.5% | 80.0% | Luminal | 97.5% | 1 | 0.0% |
| BC6 | 3.5 | 1.5 | 5 | WT | 55.0% | 3.0% | 3.0% | 7.5% | Basal | 57.5% | 1 | 0.0% |
| BC7 | 3 | 1 | 4 | WT | 37.5% | 35.0% | 55.0% | 65.0% | Luminal | 95.0% | 1 | 9.0% |
| BC8 | 3.5 | 1.5 | 5 | WT | 22.5% | 17.5% | 42.5% | 50.0% | Luminal | 95.0% | 0 | 0.0% |
| BC9 | 0 | 0 | 0 | **Mut** | 40.0% | 22.5% | 17.5% | 20.0% | Basal | 92.5% | 3 | 6.0% |
| BC10 | 4 | 1.5 | 5.5 | WT | 32.5% | 1.5% | 5.0% | 75.0% | Luminal | 97.5% | 2 | 0.0% |
| BC11 | 4.5 | 1.5 | 6 | WT | 75.0% | 45.0% | 40.0% | 42.5% | Basal | 92.5% | 1 | 17.5% |
| BC12 | 4.5 | 1.5 | 6 | WT | 7.5% | 30.0% | 22.5% | 22.5% | Basal | 100.0% | 2 | 0.0% |
| BC13 | 3 | 3 | 6 | WT | 30.0% | 20.0% | 32.5% | 45.0% | Luminal | 87.5% | 2 | 0.0% |
| BC14 | 5 | 2.5 | 7.5 | **Mut** | 90.0% | 17.5% | 65.0% | 65.0% | Basal | 98.5% | 1 | 3.0% |
| BC15 | 4 | 2 | 6 | WT | 20.0% | 15.0% | 45.0% | 50.0% | Luminal | 97.5% | 3 | 0.0% |

**Supplementary Table S12.** Differentially expressed urinary miRNAs between luminal type and basal type bladder cancer patients.

| miRNA | Base mean | Differential expression | log2 Fold Change | Adjusted p value |
| --- | --- | --- | --- | --- |
| hsa-miR-204-5p | 1306.403 | Downregulated | -3.94645 | 9.13E-07 |
| hsa-miR-615-3p | 26.76087 | Downregulated | -3.96412 | 6.63E-05 |
| hsa-miR-185-5p | 3655.865 | Upregulated | 3.474965 | 0.000155 |
| hsa-miR-10b-5p | 33918.78 | Downregulated | -2.80901 | 0.000155 |
| hsa-miR-30c-5p | 5294.526 | Downregulated | -1.93462 | 0.002347 |
| hsa-miR-652-3p | 283.2014 | Upregulated | 2.017157 | 0.005294 |
| hsa-miR-378c | 2120.187 | Upregulated | 2.175986 | 0.005965 |
| hsa-miR-196a-5p | 278.1883 | Downregulated | -2.22854 | 0.006362 |
| hsa-miR-30a-3p | 1178.644 | Downregulated | -2.39381 | 0.007424 |
| hsa-miR-514a-3p | 180.5613 | Downregulated | -3.01643 | 0.007424 |
| hsa-miR-17-5p | 1639.516 | Upregulated | 2.775495 | 0.008161 |
| hsa-miR-576-3p | 106.3659 | Upregulated | 1.973681 | 0.010475 |
| hsa-miR-629-5p | 309.0403 | Upregulated | 1.704317 | 0.01686 |
| hsa-miR-222-3p | 2700.546 | Downregulated | -1.8818 | 0.02266 |
| hsa-miR-210-3p | 2007.653 | Upregulated | 2.091053 | 0.023872 |
| hsa-miR-30a-5p | 13298.58 | Downregulated | -1.76725 | 0.024095 |
| hsa-miR-192-5p | 5575.114 | Downregulated | -1.59323 | 0.027663 |
| hsa-miR-22-5p | 350.5727 | Downregulated | -1.72736 | 0.027978 |
| hsa-miR-93-5p | 2262.496 | Upregulated | 2.310866 | 0.028174 |
| hsa-miR-19b-3p | 453.1706 | Upregulated | 1.590633 | 0.036448 |
| hsa-miR-187-3p | 464.6924 | Upregulated | 2.800588 | 0.041854 |
| hsa-miR-455-5p | 200.753 | Downregulated | -1.81149 | 0.045525 |
| hsa-miR-23a-3p | 2887.333 | Upregulated | 1.671606 | 0.045525 |
| hsa-miR-21-5p | 147900.9 | Downregulated | -1.17157 | 0.046368 |
| hsa-miR-425-3p | 151.4769 | Upregulated | 1.5795 | 0.046368 |

**Supplementary Table S13**. The diagnostic ability to distinguish luminal type and basal type bladder cancer with the three classification algorithms (naïve Bayes, logistic regression, and random forest) run on datasets consisting of top three differentially expressed miRNAs alone (miR-204-5p, miR-615-3p, and miR-185-5p), SERS data alone, or a combination of the two (miRNA + SERS).

| Model | Input | AUC | CA | F1 | Precision | Recall |
| --- | --- | --- | --- | --- | --- | --- |
| Logistic Regression | miRNA | 0.93 | 0.85 | 0.85 | 0.85 | 0.85 |
|  | SERS (PC8) | 0.98 | 0.92 | 0.92 | 0.93 | 0.92 |
|  | miRNA + SERS | 0.98 | 0.92 | 0.92 | 0.93 | 0.92 |
| Naïve Bayes | miRNA | 0.86 | 0.77 | 0.77 | 0.77 | 0.77 |
|  | SERS (PC8) | 0.91 | 0.92 | 0.92 | 0.93 | 0.92 |
|  | miRNA + SERS | 0.95 | 0.85 | 0.85 | 0.85 | 0.85 |
| Random Forest | miRNA | 0.88 | 0.69 | 0.69 | 0.69 | 0.69 |
|  | SERS (PC8) | 0.88 | 0.85 | 0.85 | 0.85 | 0.85 |
|  | miRNA + SERS | 0.93 | 0.77 | 0.77 | 0.77 | 0.77 |

AUC = Area under the ROC curve; CA = classification accuracy; F1 = harmonic mean of precision and recall.

# **References**

1. Li M*, et al.* (2015) Reagent- and separation-free measurements of urine creatinine concentration using stamping surface enhanced Raman scattering (S-SERS). *Biomed Opt Express* **6:** 849-858.

2. Westley C*, et al.* (2017) Absolute Quantification of Uric Acid in Human Urine Using Surface Enhanced Raman Scattering with the Standard Addition Method. *Anal Chem* **89:** 2472-2477.

3. Gurian E*, et al.* (2020) Differentiation between stages of non-alcoholic fatty liver diseases using surface-enhanced Raman spectroscopy. *Anal Chim Acta* **1110:** 190-198.

4. Huang Z*, et al.* (2021) Correlation of surface-enhanced Raman spectroscopic fingerprints of kidney transplant recipient urine with kidney function parameters. *Sci Rep* **11:** 2463.

5. Casella M*, et al.* (2011) Raman and SERS recognition of beta-carotene and haemoglobin fingerprints in human whole blood. *Spectrochim Acta A Mol Biomol Spectrosc* **79:** 915-919.
